# Prevalence and Predictors of Mild Cognitive Impairment in Xi’an: A Community-Based Study among the Elders

**DOI:** 10.1371/journal.pone.0083217

**Published:** 2014-01-08

**Authors:** Xiangni Su, Lei Shang, Qiaoling Xu, Nannan Li, Jianhua Chen, Liping Zhang, Lei Zhang, Qianzhen Hua

**Affiliations:** 1 Department of Nursing, the Fourth Military Medical University, Xi’an, China; 2 Department of Health Statistics, the Fourth Military Medical University, Xi’an, China; 3 Department of Nursing, the Health College, Xi’an, China; 4 Department of Epidemiology, the Fourth Military Medical University, Xi’an, China; Oregon Health & Science University, United States of America

## Abstract

Mild cognitive impairment (MCI) is an intermediate stage between normal cognitive function and dementia among aging individuals. This study was designed to estimate the prevalence of MCI and explore the possible risk factors including gender disparities among community-dwelling older individuals. The study was conducted in Xi’an, China. This is a cross-sectional study. A total of 815 individuals, 60 years and older were selected by stratified random cluster sampling. Cognitive function was measured using the mini-mental status examination (MMSE), the Chinese version of the Dementia Rating Scales (CDRS) was used to apply the diagnostic of non-dementia, and activities of daily living (ADL) and instrumental activities of daily living (IADL) systems were used to functional status. The association between sociodemographic characteristics, lifestyle, history of chronic diseases and MCI were evaluated separately for men and women using the Pearson χ^2^- test and binary logistic regression. Of the 815 community-dwelling individuals, 145 were found to have MCI. Overall, the prevalence of MCI was 18.5%, with a prevalence of 19.6% in women (105/535), and 15.3% (40/261) in men. The results of the binary logistical regression analysis indicated that age and history of stroke were associated with MCI in men. For women, the risk factors were lower level of educational and lack of religious attendance. Results suggested that the factors capable of influencing MCI differed profoundly between older men and older women. For this reason, different preventative measures should be adopted to delay or reverse cognitive impairment among community-dwelling older men and women.

## Introduction

The prevalence of degenerative dementias and other conditions associated with Alzheimer’s disease is increasing due to the rapid aging of the population. The prevalence is below 1% among people aged 60–64 years, but it shows an almost exponential increase with age. In western countries, the prevalence ranges from 24%–33% for people aged 85 years or older. It has been reported that Chinese dementia patients make up 40% of all dementia patients in the Asia-Pacific region and 25% of dementia patients globally [1]. There are 6–7 million Chinese people with dementia, with an incidence of 5–7% among people over 65 years of age [2]. Dementia patients experience not only a serious decline in individual quality of life but also impose a heavy economic burden on their families and society. In Mainland China, dementia is responsible for an estimated 51.3–59.8 billion yuan in annual healthcare costs.

Mild cognitive impairment (MCI) is an intermediate stage between normal cognitive function and dementia among the older population [3]. People with MCI appear to have a significantly higher risk of dementia. It is reported that 10–15%, 60.5% and 100% of MCI patients will develop full dementia within 1 year, 5 years and 9.5 years, respectively, after initial diagnosis with MCI [4]. The probability of death or progression to dementia for an elderly person within five years of diagnosis of MCI is 2.2 times and 3.26 times higher, respectively, than that of an older individual without MCI. The probability that an MCI patient will develop dementia within 10 years of initial diagnosis is 4.35 times that of a similar individual without MCI [5]. In China, the number of people aged 60 years and older has increased sharply from 130.0 million in 2000 to 181.7 million in 2010. This accounts for 13.26% of the total population. Given a typical family size of five people spanning three generations, at least 30–35 million people are directly or indirectly affected by the deleterious effects of dementia in China. Moreover, family-based assistance for dementia patients is less, with only 2% of families being capable of offering care for dementia patients [2]. Therefore, dementia has become a major public health problem in China. Early identification of subjects at risk of MCI and adopting effective preventive strategies can delay or prevent dementia.

Some studies have shown that the factors related to MCI include age, sex, diet, lifestyle, and chronic diseases [6]. However, few studies have evaluated the effects of MCI on community-dwelling older people, and none of the studies that were performed in China stratified results by gender. The objective of the present study was to investigate the prevalence of MCI among community-dwelling older subjects and to evaluate the effects of MCI among individuals of different genders to outline the impact of gender disparities and any differential effects on the occurrence of MCI in Xi’an. These results might provide valuable reference material for a preventive strategy capable of reducing the incidence of dementia in China.

## Materials and Methods

### 1. Calculation of Sample Size

The reported prevalence of MCI (*P_exp_*) ranges from 10%–20% [7]–[11]. The required sample size (*N*) was calculated to obtain a 95% confidence interval (α = 0.05) was determined using the following formula:
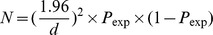



Here, *d* is the desired absolute precision. It was assumed that *d* = ±3%. The range of the required sample size was between 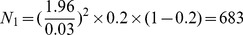 and 
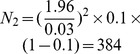
. We select *N*_1_ and considering the no response subjects, we added 15% subjects; therefore, we determined that the sample size for the present study should be no less than 785. The sample size of *N* = 815 were chosen for the present study.

### 2. Subjects

Xi’an is located in western China. According to the 2010 Chinese census, the city had a population of 8.41 million people. There are 6 districts and 760 communities, including urban and rural areas. For the present study, 11 communities were randomly selected using a cluster sampling method. All the elderly individuals aged 60 years and older who were living in the selected communities were invited to answer a standardized questionnaire. Individuals were excluded if they had lived in the selected community for less than five years or were not permanent residents, did not agree to take part in this study, had severe cognitive impairment, or if they were otherwise unable to communicate with investigators normally. A total of 815 subjects were selected to participate in this study, and 796 subjects completed the questionnaires thoroughly (response rate 97.7%).

This study was approved by the Ethics Committee of the Fourth Military Medical University Ethics Committee. Participants provided written informed consent prior to the survey.

### 3. Definition of MCI

This study adopted MCI diagnostic criteria from Petersen definitions with two modifications [12]: (I) A combination of activities of daily living (ADL) and instrumental activities of daily living (IADL) was used to define intact ADL; (II) (1) memory complaints (either self-reported or reported by family members or caregivers); (2) continued normal cognitive function, as assessed through the mini-mental state examination (MMSE) [13]; (3) essentially intact ADL [14] and IADL [15]; (4) no clear dementia (≤1.5 S.D. form norm), as evaluated by the Chinese version of Dementia Rating Scale (CDRS) [16]; (5) no abnormal memory impairment for age.

### 4. Survey

#### 4.1 Cognitive performance

The mini-mental state examination (MMSE) is the most commonly used method of screening for cognitive impairment and dementia worldwide [17]. In the present study, cognitive function was assessed using the Chinese version of MMSE score with the subjects’ education taken into account diagnosed as MCI if MMSE≤17 for illiterates; MMSE ≤20 for primary school graduates (≥6 years of education), MMSE≤24 for junior school graduates or above (≥9 years of education) [18]–[19]. The Chinese version of the MMSE has a high sensitivity (90.8%) and specificity (93%) in the detection of dementia within the Chinese populations [20]. It covers the following five areas of cognition: orientation, memory, attention and calculation, recall and language [21]. Respondents were asked 5 orientation-related questions (the current time, place, season, community and the name of the county), 1 memory questions (3 words were mentioned and respondents were asked to repeat them once and remember them), 5 attention and calculation questions (respondents were asked to serially subtract 7 from 100), 1 recall question (respondents were asked to repeat the 3 words from the memory question), 3 language questions (respondents were asked to repeat a sentence and name simple items, such as pens and watches, which were shown to them), 1 drawing question, and 3 comprehension questions (respondents were asked to take a piece of paper in their right hand, fold it, and then place it on their left leg). Responses to the questions were categorized as “incorrect answer”, given 0 point, and “correct answer” given 1 point. The final score ranged from 0 to 30. High values indicated better cognitive function.

#### 4.2 Sociodemographic information form

A sociodemographic information form was designed by the research group. The form included gender (man, woman), age (years), ethnicity (Han, Hui), marital status (with or without spouse), living arrangements (defined as alone, with spouse, children or others), education (years of schooling), monthly income (RMB) (less than 1000 yuan; 1000–1999 yuan; 2000 yuan and above), religious attendance (Buddhism, Christianity, Islam), smoking, drinking, physical activity (everyday, 3–5 times per week, 1–2 times per week and never), Social activity was assessed based on questions about participation in religious, community and social activities and contact with friends, relatives and neighbors, history of chronic disease (hypertension, cardiovascular disease, stroke, cancer, chronic disease of respiration system), height, weight, and other common factors.

Height and weight were measured by the investigators using standard methods. The weight status was defined by BMI as follows: underweight, below 18.5 kg/m^2^; normal weight, 18.5–23.9 kg/m^2^; overweight, 24–27.9 kg/m^2^ and obese, 28 kg/m^2^ or heavier [22].

#### 4.3 Functional status

Functional status was assessed by using a 6 item on the activities of daily living (ADL) scale [14] and a 5 items of instrument activities of daily living (IADLs) [15]. Both scales have been widely used in the literature and both were later adapted by Elena Yu and William Liu and used in a study on Chinese patients [23]. The Chinese version of the ADL scale includes 20 items and its reliability and validity have been verified in older Chinese people [24]. Each item is independently coded from 1 (independent) to 4 (totally dependent). A person is considered dependent in an activity of daily living as soon as the code for the corresponding item exceeds 1. The sum of the 20 items constitutes the ADL scores ranging from 20 (maximum independence) to 80 (maximum dependence).

#### 4.4 Non-dementia performance

The Chinese version of the Mattis Dementia Rating Scale (CDRS) was used to assess the general cognitive status, and has been considered by many Chinese researchers to be a very useful instrument for rating patients with dementia and therefore has frequently been used both in clinical practice and in research [25]–[26]. It can measure attention, initiation and perseveration, construction, conceptualization and memory. The CDRS cut-off scores for dementia in the Chinese population according to education level, illiteracy, primary school graduates (≥6 years of education), junior school graduates (≥9 years of education) revealed a sensitivity was 85%, 94%, 94%, respectively, and a specificity of 90%, 94%, 92%, respectively [27].

#### 4.5 Data collection and quality control

Four graduate students were selected from the FMMU School of Nursing. All were trained in the use of normal surveys to ensure that they understood the purpose and requirements of the present study. Face-to-face interviews were conducted. An investigator explained the purpose and details of the questionnaire so that each subject could understand every item. After the investigator explained an item, the subject selected one answer according to his/her actual status, and the investigator recorded it on the questionnaire. One of the main investigators carefully rechecked the questionnaires. Telephone interviews were conducted for any lost or incomplete information. To ensure a response rate over 95%, investigators performed general physical examinations and provided gifts to subjects who completed the questionnaires thoroughly. Data from valid questionnaires were entered into an Epidata 3.1 software database. To ensure accuracy, double entry mode was selected and a logic check for errors was performed.

#### 4.6 Statistical analysis

Quantitative data were expressed as 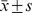, and qualitative data were also expressed as percentages. According to various categorical variables, the Pearson χ^2^-test was used to compare differences in the prevalence of MCI. Logistic regression was used to examine the risk factors for MCI among men and women with MCI (no = 0, yes = 1) as the dependent variable. Age (years), level of education (no formal education = 1, primary school = 2, junior school = 3, senior school = 4, college and above = 5), stroke (no = 0, yes = 1), and religious attendance (no = 0, yes = 1) severed as independent variables. *P<*0.05 indicated statistical significance. All analyses were performed using the Statistical Package for Social Sciences (version 17.0; SPSS Inc., Chicago, IL, US).

## Results

### 1. General Characteristics of Subjects

Demographic information of the subjects is presented in Table 1. A total of 796 individuals aged 60–86 years completed the questionnaires. Of these 62.7% were women and 32.8% were men, with a mean age of 71.4±6.8 years. The prevalence of MCI was 18.2%, 15.3% for men and 19.6% for women. The rates of smoking and alcohol consumption were 5.9% and 7.0%, respectively. The proportion of widows, lower levels of education, and lower monthly household incomes were all higher among women respondents (*P*<0.01). Women were more likely to attend religious services than men (*P*<0.01). Men tended to consume more alcohol (*P*<0.01). Approximately 85.1% of the subjects lived under the same roof as their spouse, children, or others companions, and the difference was significant (*P = *0.020). The prevalence of hypertension, cardiovascular disease, stroke, cancer and respiratory disease were similar between male and female participants (*P*>0.05).

**Table 1:** Demographics and history of chronic diseases among men and women respondents^a^ (n = 796) n (%).

Parameters	Men (n = 261)	Women (n = 535)	Total (n = 796)
MCI	40(15.3)	105(19.6)	145(18.2)
Age^b^			
60–77	193(73.9)	432(80.7)	625(78.5)
78 and older	68(26.1)	103(19.3)	171(21.5)
Educational status^b^			
No formal education(0)	10(3.8)	99(18.5)	109(13.7)
Primary school (1–6)	51(19.5)	139(26.0)	190(23.9)
Junior high school (7–9)	85(32.6)	164(30.7)	249(31.3)
Senior high school/technology (10–12)	59(22.6)	108(20.2)	167(21.0)
College or above (≥13)	56(21.5)	25(4.7)	81(10.2)
Income (yuan/month)^b^			
Less than1000	9(3.4)	111(20.7)	120(15.1)
1000–1999	50(19.2)	278(52.0)	328(41.2)
2000 or above	202(77.4)	146(27.3)	348(43.7)
Spouse^c^			
With spouse	223(85.4)	310(57.9)	533(67.0)
Without spouse	38(14.6)	225(42.1)	263(33.0)
Living arrangement^b^			
Alone	28(10.7)	91(17.0)	119(14.9)
With spouse/children/others	233(89.3)	444(83.0)	677(85.1)
Religious attendance^b^	19(7.3)	98(18.3)	117(14.7)
Weight status			
Underweight (BMI <18.5 kg/m^2^)	12(4.6)	20(3.7)	32(4.0)
Normal (BMI: 18.5–24 kg/m^2^)	106(40.6)	213(39.8)	319(40.1)
Overweight (BMI: 24–28 kg/m^2^)	113(43.3)	212(39.6)	325(40.8)
Obese (BMI >28 kg/m^2^)	30(11.5)	90(16.8)	129(15.1)
Smoking	18(.9)	38(7.1)	56(7.0)
Alcohol consumption^b^	37(4.2)	10(1.9)	47(5.9)
Hypertension	106(40.6)	238(44.5)	344(43.2)
Cardiovascular disease^b^	54(20.7)	148(27.7)	202(25.4)
Stroke	54(20.7)	97(18.1)	151(19.0)
Cancer	4(1.5)	6(1.1)	10(1.3)
Chronic disease of respiration system	35(13.4)	64(12.0)	99(12.4)

^a^ *χ* ^2^ *-*test or Fisher test were used.

b significance at *P*<0.05.

### 2. Mini-mental Status Examine of Performance by Gender Disparities

Generally, men performed better than women in neuropsychological assessment (Table 2). There were significant differences in the MMSE scores (*P*<0.0001), orientation (*P*<0.0001), attention and calculation (*P*<0.0001), and language (*P*<0.0001) between men and women, but no significant difference with respect to memory or recall.

**Table 2:** Scores in various domains of MMSE for men and women^a^ (n = 796).

Neuropsychologicaltest	Men (n = 261)	Women (n = 535)	Total (n = 796)
MMSE	27.38±2.67	25.84±4.18^b^	26.35±3.82
Orientation	9.75±0.71	9.34±1.31^b^	9.47±1.17
Memory	2.87±0.50	2.79±0.68	2.82±0.63
Attention and calculation	4.13±1.33	3.60±1.66^b^	3.78±1.58
Recall	1.95±1.04	1.94±1.05	1.94±1.05
Language	8.68±0.68	8.17±1.24^b^	8.34±1.12

a student *t*-test was used.

b *P*<0.01: males compared to females.

### 3. Factors Associated with MCI Incidence between Gender Disparities

The results of χ^2^ -testing showed that advanced age and history of stroke were significantly associated with MCI among men. However, educational status and lack of religious attendance were found to be associated with the occurrence of MCI among women (Table 3).

**Table 3:** Factors associated with MCI among men and women (%).

	N	Men (n = 261)		Women (n = 535)	
Factors	796	N-MCI	MCI	N-MCI	MCI
Ethnicity					
Han Chinese	777(97.6)	218(84.8)	39(15.2)	417(80.2)	103(19.8)
Hui Chinese	19(2.4)	3(75.0)	1(25.0)	13(86.7)	2(13.3)
Age^a/c^					
60–77	625(78.5)	170(88.1)	23(11.9)	348(80.6)	84(19.4)
78 or older	171(21.5)	51(75.0)	17(25.0)	82(79.6)	21(20.4)
Spouse^c^					
With spouse	533(67.0)	189(85.4)	34(14.6)	253(81.6)	57(18.4)
Without spouse	263(33.0)	32(85.0)	6(15.0)	177(78.7)	48(21.3)
Years of educationb,c					
No formal education(0)	109(13.7)	6(60.0)	4(40.0)	79(79.8)	20(20.2)
Primary school(1–6)	190(23.9)	43(84.3)	8(15.7)	100(71.9)	39(28.1)
Junior high school(7–9)	249(31.3)	73(85.9)	12(14.1)	134(81.7)	30(18.3)
Senior high school/technical school (10–12)	167(21.0)	46(78.0)	13(22.0)	95(88.0)	13(12.0)
College or above(≥13)	81(10.2)	53(94.6)	3(5.4)	22(88.0)	3(12.0)
Living arrangements					
Alone	677(85.1)	198(85.0)	35(15.0)	73(80.2)	18(19.8)
With spouse/children/others	119(14.9)	23(82.1)	5(17.9)	357(80.4)	87(19.6)
Religious attendanceb,c	117(14.7)	16(84.2)	3(15.8)	67(68.4)	31(31.6)
Monthly income^c^					
Below 1000	120(15.1)	9(100.0)	0(0.0)	90(81.1)	21(18.9)
1000–1999	328(41.2)	43(86.0)	7(14.0)	219(78.8)	59(21.2)
2000 or higher	348(43.7)	169(83.7)	33(16.3)	121(82.9)	25(17.1)
Hypertension	344(43.2)	136(87.7)	19(12.3)	188(79.0)	50(21.0)
Alcohol consumption^c^	47(5.9)	34(91.9)	3(8.1)	10(100)	0(0.0)
Smoking status	56(7.0)	16(88.9)	2(11.1)	30(78.9)	8(21.1)
Cardiovascular disease	202(25.4)	42(77.8)	12(22.2)	122(82.4)	26(17.6)
Stroke^a/c^	151(19.0)	37(68.5)	17(31.5)	80(82.5)	17(17.5)
Cancer	10(1.3)	4(100.0)	0(0.0)	4(66.7)	2(33.3)
Respiratory disease	99(12.4)	32(91.4)	3(8.6)	54(84.4)	10(15.6)
Exercise					
Every day	164(20.6)	52(83.9)	10(16.1)	86(84.3)	16(15.7)
3–5 times per week	334(42.0)	78(81.3)	18(18.8)	192(80.7)	46(19.3)
1–2 times per week	230(30.7)	70(87.5)	10(12.5)	114(76.0)	36(24.0)
Never	68(8.8)	21(91.3)	2(8.7)	38(84.4)	7(15.6)
Participation in organized group Social activity					
Never	225(28.3)	13(92.9)	1(7.1)	35(77.8)	10(22.2)
Occasionally	59(7.4)	48(80.0)	12(20.0)	132(80.0)	33(20.0)
Often	393(49.4)	127(85.8)	21(14.2)	199(81.2)	46(18.8)
Every day	119(14.9)	33(84.6)	6(15.4)	64(80.0)	16(20.0)
ADL disability	85(10.7)	23(88.5)	3(11.5)	45(76.3)	14(23.7)
IADL disability	173(21.7)	36(80.0)	9(20.0)	101(78.9)	27(21.1)
BMI^c^					
Underweight (BMI <18.5 kg/m^2^)	32(4.0)	9(75.0)	3(25.0)	18(90.0)	2(10.0)
Normal (BMI: 18.5–24 kg/m^2^)	319(40.1)	87(82.1)	19(17.9)	177(83.1)	36(16.9)
Overweight (BMI:24–28 kg/m^2^)	325(40.8)	97(85.8)	16(14.2)	171(80.7)	41(19.3)
Obese (BMI>28 kg/m^2^)	120(15.1	28(93.1)	2(6.9)	64(71.1)	26(28.9)

a Factors were associated with MCI among men (*P*<0.05).

b Factors were associated with MCI among women (*P*<0.05).

c There were significant differences in prevalence rates of MCI between men and women (*P*<0.05).

Binary logistic regression analysis also showed that advanced age (OR = 2.358; *CI = *1.099–5.060; *P = *0.028) and history of stroke (OR = 3.814; *CI = *1.766–8.235; *P = *0.001) were related to MCI among men. However, higher education (OR = 2.870; *CI = *0.773–10.655; *P = *0.032) and lack of religious attendance (OR = 0.430; *CI = *0.252–0.732; *P = *0.002) were found to be significantly associated with MCI among women (Table 4).

**Table 4:** Predictors of MCI by gender disparities.

predictor	OR (95% *CI*)	Wals	*P* a
Male			
Age	2.358(1.099–5.060)	4.852	0.028^b^
Stroke	3.814(1.766–8.235)	11.621	0.001^b^
Women	OR (95% *CI*)	Wals	*P*
Years of education	0.782(0.622–0.983)	4.431	0.035^b^
Religious attendance	0.430(0.252–0.732)	9.049	0.003^b^

a Data were analyzed using binary logistic regression; ^b^Significance at *P*<0.05.

## Discussion

Mild cognitive impairment (MCI) is an intermediate stage between normal cognition and dementia among aging individuals. Early detection of MCI is helpful in the prevention of dementia. The results of the present work showed the prevalence of MCI among older individuals in Xi’an to be 18.2%, 15.3% in men and 19.6% in women. Advanced age and history of stroke were the main predictors for MCI in men, and years of education and lack of religious attendance were the significant predictors for MCI in female.

The prevalence of MCI in the general aging population (older than 65 years) has been reported to be 3.1% in the United States and 4.9% in Japan [28]–[29]. A systematic analysis of 22 studies in China described a pooled prevalence of MCI of 12.7% among older individuals [30]. Similar studies conducted in Beijing [31] and Guangzhou [8] showed the prevalence of MCI to be 8.9% and 5.47%, respectively. Due to the operational diagnostic criteria, disparate assessment procedures, geographical boundaries and participant backgrounds, the prevalence of MCI was found to be higher in the present study than in other studies. Regardless of gender disparities, the prevalence of MCI has been shown to be on an upward trend in urban areas of China.

As expected, a completely different conceptual factor capable of predicting MCI was observed in men and women. In the present study, age was found to be associated with MCI among men, but not among women. Similarly, the results of a study examining the causes of cognitive impairment showed aging to be the predominant risk factors MCI and also showed the prevalence of MCI to increase with age [32]. Some studies have also shown the prevalence of MCI to be higher in people 75 years of age and older than among those younger than 75 years [8], [33]. Controversially, other results have indicated no significant relationship between age and MCI [34]. However, none of these studies have evaluated the effects of gender perseverance. Instead, they suggested an overall effect of aging on MCI. At the moment, the mechanisms underlying this connection may include the fact that aging produces free radical damage, oxidative stress, alterations in calcium homeostasis, and endothelial damage, which coincide with the reduced efficacy of amyloid clearance and increases the likelihood of cerebrovascular disease. This causes synaptic damage, loss of transmitters and receptors, and inflammation, which results in neuronal death and subsequent clinical symptoms. Many of these processes are partially understood, and therapeutic options have been proposed for some of them. The present study supports the hypothesis that age is a risk factor for MCI.

The present study also demonstrated that a history of stroke is associated with cognitive impairment among men. These findings are similar to those of a previous study among older residents of Beijing, China [31]. That study indicated that stroke and hypertension are independent risk factors for MCI among older individuals. An extensive systematic review showed the prevalence of dementia among individuals who had sustained clinical strokes to be 10% prior to the first stroke with an additional 10% of developing new dementia shortly after the first stroke [35]. The occurrence of a second stroke is a powerful predictor of dementia. Among individuals with recurrent stroke the prevalence of dementia was 30%. Although cardiovascular risk factors were excluded, the relationship of the risk of MCI to stroke remained the same [31]. Even when cerebrovascular injury does not cause measurable deficits itself, it can still exacerbate cognitive dysfunction in the presence of a concomitant degenerative process. For example, preexisting brain damage attributable to stroke could additionally increase the likelihood of dementia by increasing the extent of an injury from the molecular and cellular cascade of dementia. For this reason, it is important to prevent and cure cerebrovascular disease.

The present study we also demonstrated a lack of formal educational experience to be associated with MCI in women. Women who only had an elementary school education were more likely to develop MCI than those with higher levels of education. This was consistent with studies that have shown education to have a protective effect with respect to dementia [36]–[37]. However, some studies have shown that education does not have a protective effect against dementia [38]–[39]. The cognitive reserve hypothesis postulates that a higher level of education increases neuronal plasticity and connectivity. However, gender a confounding variable was not taken into account in any previous study. In the present investigation, a number of older women had no formal education and lacked of social contacts (visiting friends, entertaining guests). These phenomena were extremely closely associated with traditional Chinese culture. For such women, certain measures such as reading books, writing, and taking part in classes for seniors may help prevent MCI.

The present study also demonstrated a trend towards an increasing risk of MCI in women who did not attend religious services or participate in religious organizations. This is one of the first studies to examine the effects of religious attendance and religious identity on cognitive functioning [40]. Van Ness et al found religious attendance rather than religious identity to be associated with a reduction in the risk for cognitive dysfunction in a 6-year study. Another study evaluated whether religious attendance was associated with slower rates of cognitive decline [41]. Using a four-wave data set collected from a sample of 3050 older Mexican-American individuals, Hill et al constructed a series of linear growth curve models showing religious attendance to be associated with lower rates of cognitive decline among older Mexican-Americans over an 8- year study. However, results of these studies did not take gender into consideration. Results have shown religious participation to be significantly associated with lower odds of cognitive impairment for both sexes, but there were more cognitive benefits for men than for women [42]. For many women, church services may offer networking opportunities, structure and organization, and a sense of purpose [43]. Some studies have found women to be more likely than men to use religion as a form of consolation or coping method when faced with health problems [44]. These results should be taken into account when developing preventive strategies meant for community-dwelling older individuals.

The present study has several limitations. First, it was a cross-sectional study and therefore, cannot provide evidence of any causal relationship between predictors and MCI. Second, only a few tests and scales were used, which may increase the number of false positive results for participants with fewer years of education. Third, education may affect the results of some screening tests, including the MMSE and rates of MCI, which may be overestimated among illiterate individuals. However, this is the first study to specifically investigate the effects of gender disparities on the risk of MCI among the community-dwelling older population in Xi’an, and participants with dementia or severe cognitive impairment at baseline were excluded. None of participants were institutionalized. For these reason, these results can be readily extrapolated to the older population of China in general.

## Conclusion

The results of the present study demonstrated a high prevalence (18.5%) of MCI among the community-dwelling older individuals of Xi’an. Advanced age and stroke were found to be associated with cognitive impairment among men, and lower levels of education and lack of religious attendance were found to be associated with MCI among women. This indicates that the role of gender disparities governing the occurrence of MCI merits further investigation in this unique population. Identifying individuals at high risk for MCI is crucial. This may be helpful in the preventing MCI. They may also be useful in developing intervention, many of which have been conducted to delay the progression of MCI to its major proposed endpoint, Alzheimer’s disease (AD).

## References

[1] Anon (2009) There will be over 35 million patients with elderly dementia in the globe. Avaliable: http://www.ebiotrade.com. Accessed 22 September 2010.

[2] Zhou SN, Liu KB (2006) The progress of gerontic dementia in diagnosis and treatment. Chin Stroke 10: 741–748.

[3] Petersen RC, Smith GE, Waring SC, Ivnik RJ, Tangalos EG, et al (1999) Mild cognitive impairment: clinical characterization and outcome. Arch Neurol 56: 303–308. 10190820 10.1001/archneur.56.3.303

[4] Morris JC, Storandt M, Miller JP, McKeel DW, Price JL, et al (2001) Mild cognitive impairment represents early-stage Alzheimer disease. Arch Neurol 58: 397–405. 11255443 10.1001/archneur.58.3.397

[5] Zhu ZQ, Li CB, Zhang MY (2001) Prognosis and sequelae of mild cognitive impairment in the community’s elderly people. Shanghai Arch Psychiatry 13: 12–14.

[6] Fernandez MM, Castro FJ, Perez DLHS, Mandaluniz LA, Gordejuela MM, et al (2008) Risk factors for dementia in the epidemiological study of Munguialde County (Basque Country-Spain). BMC Neurol 8: 39. 18922150 10.1186/1471-2377-8-39 PMC2584030

[7] Xu MG, Li CH-B, He YL, Hui Z (2001) A preliminary study of the epidemiology of successful aging and mild cognitive impairment in community elderly. Shanghai Arch Psychiatr 13 IS (supplement): 15–18.

[8] Huang RY, Tang MN, Ma C, Guo YB, Han HY, et al (2008) The prevalence of mild cognitive impairment of residents aged 60 years and over in the urban and rural areas in Guangzhou. Chin J Nerv Ment Dis 34: 533–537.

[9] Liu JL, Gao H, Song F, Li JL, Shi W, et al (2007) Analysis of risk factors of cognitive handicap and senile dementia in honorary retired and ordinary retired cadres from 51 cadre sanatoriurns of 11areas in Yellow River Valley. J Clin Rehabilitative Tissue Eng Res 11: 5869–5871.

[10] Wu YG, Li ZH-B, Zhang GB, Zhang LG, Xu ZY, et al (2005) Apreliminary study of the epidemiology of succeddful aging, usual aging and mild cognitive impairment in Bma elderly. J Guangxi Med Univ 22: 366–367.

[11] Lei MY, Huang W, Yang JY, Gao LY, Wei LF, et al.. (2008) Prevalence of mild cognitive impairment old people in urban and rural areas of Guizhou province. Chin Ment Health J 22.

[12] Petersen RC (2004) Mild cognitive impairment as a diagnostic entity. J Intern Med 256: 183–194. 15324362 10.1111/j.1365-2796.2004.01388.x

[13] Folstein MF, Folstein SE, McHugh PR (1975) “Mini-mental state”. A practical method for grading the cognitive state of patients for the clinician. J Psychiatr Res 12: 189–198. 1202204 10.1016/0022-3956(75)90026-6

[14] Katz S, Ford AB, Moskowitz RW, Jackson BA, Jaffe MW (1963) Studies of illness in the aged, the index of ADL: a standardized measure of biological and psychosocial function. J Am Geriatr. 37: 267–271. 10.1001/jama.1963.03060120024016 14044222

[15] Lawton MP, Brody EM (1969) Assessment of older people: self-maintaining and instrumental activities of daily living. Gerontologist 9: 179–186. 5349366

[16] Mattis S (1988) Dementia Rating Scale. Profession Manual. Florida: Psychological Assessment Resource.

[17] Shulman KI, Herrmann N, Brodaty H, Chiu H, Lawlor B, et al (2006) IPA survey of brief cognitive screening instruments. Int Psychogeriatr 18: 281–294. 16466586 10.1017/S1041610205002693

[18] Cui GH, Yao YH, Xu RF, Tang HD, Jiang GX, et al (2001) Cognitive impairment using education-based cutoff points for CMMSE scores in elderly Chinese people of agricultural and rural Shanghai China. Acta Neurol Scand 124: 361–367. 10.1111/j.1600-0404.2010.01484.x 21303351

[19] Katzman R, Zhang MY, Ouang-Ya-Qu, Wang ZY, Liu WT, et al.. (1988) A Chinese version of the Mini-Mental Status Examination; impact of illiteracy in a Shanghai dementia survey. J Clin Epidemiol. 10.1016/0895-4356(88)90034-0 3193141

[20] Zhang ZX, Zahner GE, Roman GC, Liu XH, Wu CB, et al (2006) Socio-demographic variation of dementia subtypes in china: Methodology and results of a prevalence study in Beijing, Chengdu, Shanghai, and Xian. Neuroepidemiology 27: 177–187. 17035714 10.1159/000096131

[21] Zhang MY, Yu E, He YL (1995) Instruments for the epidemiological survey of dementia and their applications. China Shanghai Psychiatry 7: 3–5.

[22] Zhou B (2002) Cooperative meta-analysis group of working group on obesity in China: Prospective study for cut-off points of body mass index in Chinese adults. Chin J Epide 23: 431–434. 12667353

[23] Stemmler M, Steinwachs KC, Lehfeld H, Jentzsch J (1994) Different methodological approaches for the construction of a therapy sensitive ADL scale for the assessment of Alzheimer patients. In: Jellinger K, Ladurner G, Windisch M, editors. New trends in the diagnosis and therapy of Alzheimer’s disease. Austria: Springer-Verlag. 81–90.

[24] He YL, Zhai GY, Xiong XY, Chi YF, Zhang MY, et al (1990) Assessment of activities of daily living in the elderly. Chi J Gerontol 10: 266–269.

[25] Monsch AU, Bondi MW, Salmon DP, Butters N, Thal LJ, et al (1995) Clinical validity of the Mattis dementia rating scale in detecting dementia of the Alzheimer type. Arch Neurol 52: 899–904. 7661728 10.1001/archneur.1995.00540330081018

[26] Chan AS, Choi A, Chiu H, Lam L (2003) Clinical validity of the Chinese version of Mattis dementia rating scale in differentiating dementia of Alzheimer’s type in Hong Kong. JINS 9: 45–55. 12570357 10.1017/s1355617703910058

[27] Guo QH, HONG Z, Lv CZ, Yu H, Ding D (2004) Clinical validity of Chinese version of Mattis Dementia Rating Scale in Differentiating Dementia of Alzheimer type in Shanghai. Chin J of Clinical Psychology 3: 237–243.

[28] Ritchie K (2004) Mild cognitive impairment: an epidemiological perspective. Dialogues Clin Neurosci 6: 401–408. 22034212 10.31887/DCNS.2004.6.4/kritchie PMC3181815

[29] Sasaki M, Kodama C, Hidaka S, Yamashita F, Kinoshita T, et al (2009) Prevalence of four subtypes of mild cognitive impairment and APOE in a Japanese community. Int J Geriatr Psychiatry 24: 1119–1126. 19449451 10.1002/gps.2234

[30] Nie H, Xu Y, Liu B, Zhang Y, Lei T, et al (2011) The prevalence of mild cognitive impairment about elderly population in China: a meta-analysis. Int J Geriatr Psychiatry 26: 558–563. 20878675 10.1002/gps.2579

[31] Guan SC, Tang Z, Wu XG (2008) Investigation of the incidence of mild cognitive impairment and its risk factors in an elderly population sample in Beijing area. Chin J Cerebrovasc Dis 5: 395–398.

[32] Hardy JA, Higgins GA (1992) Alzheimer’s disease: the amyloid cascade hypothesis. Science 256: 184–185. 1566067 10.1126/science.1566067

[33] Liu B, Shao H, Peng Y (2005) The investigate and analysis of mild cognitive impairment among elder population in Shenzheng Luohu District. Journal of Guangzhou University of Traditional Chinese Medicine 22: 366–369.

[34] Yu H, Guo Z, Wang X (2011) Exploration of high risk factors in lifestyle for mild cognitive impairment in elderly people. Progress in Modern Biomedicine 10: 1885–1888.

[35] Pendlebury ST, Rothwell PM (2009) Prevalence, incidence, and factors associated with pre-stroke and post-stroke dementia: a systematic review and meta-analysis. Lancet Neurol 8: 1006–1018. 19782001 10.1016/S1474-4422(09)70236-4

[36] Brayne C, Ince PG, Keage HA, McKeith IG, Matthews FE, et al (2010) Education, the brain and dementia: neuroprotection or compensation? Brain 133: 2210–2216. 20826429 10.1093/brain/awq185

[37] Perneczky R, Wagenpfeil S, Lunnetta KL (2009) Education attenuates the effect of medical temporal lobe atrophy lobe atrophy on cognitive function in Alzheimer’s disease: the MIRAGE study. J Alzheimers Dis 17: 855–862. 19542606 10.3233/JAD-2009-1117 PMC2868929

[38] Christensen H, Anstey KJ, Parslow RA, Maller J, Mackinnon A, et al (2007) The brain reserve hypothesis, brain atrophy and aging. Gerontology 53: 82–95. 17057394 10.1159/000096482

[39] Koepsell TD, Kurland BF, Harel O, Johnson EA, Zhou XH, et al (2008) Education, cognitive function, and severity of neuropathology in Alzheimer disease. Neurology 70: 1732–1739. 18160675 10.1212/01.wnl.0000284603.85621.aa

[40] Rosario PW (2012) Ablation of thyroid remnant with 30 mCi 131I in thyroid cancer patients prepared with recombinant human TSH]. Arq Bras Endocrinol Metabol 56: 338–340. 22911289 10.1590/s0004-27302012000500011

[41] Hill TD, Burdette AM, Angel JL, Angel RJ (2006) Religious attendance and cognitive functioning among older Mexican Americans. J Gerontol B Psychol Sci Soc Sci 61: P3–P9. 16399939 10.1093/geronb/61.1.p3

[42] Zhang W (2010) Religious Participation, Gender Differences, and Cognitive Impairment among the Oldest-Old in China. J Aging Res 2010: 160294. 21152194 10.4061/2010/160294 PMC2990098

[43] Jarvis GE, Kirmayer LJ, Wienfeld M, Lasry JC (2005) Religious practice and psychological distress: the importance of gender, ethnically, and immigrant status. Transcult psychiatry 42: 657–675. 16570522 10.1177/1363461505058921

[44] Ferraro KF, Koch JR (1994) Religious and health among black and white adults: examining social support and consolation. J Sci Study Relig 33: 362–375.

